# Non-covalent cyclic peptides simultaneously targeting Mpro and NRP1 are highly effective against Omicron BA.2.75

**DOI:** 10.3389/fphar.2022.1037993

**Published:** 2022-11-02

**Authors:** Shengnan Yin, Shuang Mei, Zhiqin Li, Zhen Xu, Yuting Wu, Xiujuan Chen, Dongmei Liu, Miao-Miao Niu, Jindong Li

**Affiliations:** ^1^ Department of Pharmacy, Taizhou Hospital Affiliated to Nanjing University of Chinese Medicine, Taizhou, China; ^2^ Department of Pharmaceutical Analysis, China Pharmaceutical University, Nanjing, China; ^3^ Institute of Clinical Medicine, Department of Pharmacy, The Affiliated Taizhou People’s Hospital of Nanjing Medical University, Taizhou, China

**Keywords:** COVID-19, SARS-CoV-2, main protease, neuropilin-1, virtual screening

## Abstract

Available vaccine-based immunity may at high risk of being evaded due to substantial mutations in the variant Omicron. The main protease (Mpro) of SARS-CoV-2 and human neuropilin-1 (NRP1), two less mutable proteins, have been reported to be crucial for SARS-CoV-2 replication and entry into host cells, respectively. Their dual blockade may avoid vaccine failure caused by continuous mutations of the SARS-CoV-2 genome and exert synergistic antiviral efficacy. Herein, four cyclic peptides non-covalently targeting both Mpro and NRP1 were identified using virtual screening. Among them, MN-2 showed highly potent affinity to Mpro (*K*_d_ = 18.2 ± 1.9 nM) and NRP1 (*K*_d_ = 12.3 ± 1.2 nM), which was about 3,478-fold and 74-fold stronger than that of the positive inhibitors Peptide-21 and EG3287. Furthermore, MN-2 exhibited significant inhibitory activity against Mpro and remarkable anti-infective activity against the pseudotyped variant Omicron BA.2.75 without obvious cytotoxicity. These data demonstrated that MN-2, a novel non-covalent cyclic peptide, is a promising agent against Omicron BA.2.75.

## Introduction

As of 29 May 2022, the coronavirus disease 2019 (COVID-19) pandemic, caused by severe acute respiratory syndrome coronavirus 2 (SARS-CoV-2), has resulted in more than 598 million confirmed cases and over 6.4 million deaths worldwide, making it one of the greatest challenges that national health and economic systems around the world have ever faced (Wu et al., 2020a; World Health Organization (WHO), 2022). The scientific community has made a great effort to develop effective vaccines for COVID-19 all along, with adenoviral vectored vaccines, protein vaccines, and mRNA vaccines in widespread use (Le et al., 2020; Corchado-Garcia et al., 2021; Huang et al., 2021; Li et al., 2021; Ndwandwe and Wiysonge, 2021). However, vaccine-based immunity may fail as most vaccines target the frequently mutated spike (S) protein of SARS-CoV-2 (Mengist et al., 2021; Zhang et al., 2021). With the continuous emergence of SARS-CoV-2 variants of concern (VOCs), especially the latest epidemic variant Omicron BA.2.75, a rapid decline of vaccine-based protection has occurred, resulting in an upsurge in infections and deaths (Altmann et al., 2021; Walensky et al., 2021; Kupferschmidt, 2022; Rahimi and Abadi, 2022). Although booster vaccines might be developed for new variants, novel antivirals against specific and less mutable targets may be more successful (Juno and Wheatley, 2021).

The main protease (Mpro) of SARS-CoV-2, also known as 3-chymotrypsin-like protease (3CLpro), is one of the most attractive drug targets for COVID-19 therapy (Ullrich and Nitsche, 2020). Mpro plays a central role in processing viral replicase polyproteins in host cells infected with SARS-CoV-2 (Pillaiyar et al., 2016; Jin et al., 2020). With forming a catalytically active homodimer, Mpro cleaves at a minimum of 11 distinct cleavage sites to release mature non-structural proteins, facilitating replication of SARS-CoV-2 (Jin et al., 2020). No human proteases with similar cleavage specificity to Mpro have been known, suggesting that targeting Mpro may be of high selectivity with low off-target toxicity (Zhang et al., 2020; Chan et al., 2021). Moreover, Mpro was shown to aid in immune evasion by inhibiting both interferon production and JAK-STAT signaling, which resulted in enhanced viral replication and poor patient outcomes (Wu et al., 2020b; Fung et al., 2021). To date, some Mpro inhibitors have entered clinical trials, including peptidomimetic inhibitor lufotrelvir and orally available peptidomimetic inhibitor nirmatrelvir, both of which contain an electrophilic warhead that binds to SARS-CoV-2 Mpro by forming a covalent bond (Hoffman et al., 2020; Painter et al., 2021). However, potential toxic effects may occur in drugs acting through covalently binding (Lv et al., 2022). Notably, although mutations are a common phenomenon in viral systems, Mpro seems to be relatively tolerant of mutations near the active site (Dai et al., 2020). In light of the potential negative consequences of covalently binding and uncertain mutations, anti-COVID-19 drugs targeting Mpro remain to be developed. Several new Mpro-based strategies have great potential to circumvent the above issues, such as non-covalent Mpro inhibitors or multi-targeting drugs in combination with other conservative targets (Javed et al., 2021; Rossetti et al., 2022).

Neuropilin-1 (NRP1), a transmembrane receptor that is conservative compared to SARS-CoV-2, has been identified as a host mediator for SARS-CoV-2 cell entry and infection (Cantuti-Castelvetri et al., 2020; Daly et al., 2020; Baindara et al., 2022). The b1 domain of NRP1 (NRP1-BD) can be bound and activated by the amino acid sequence RRAR (where R is arginine, and A is alanine) of the C-end arginine-rich portion of SARS-CoV-2 S1 protein, which conforms to the “C-end rule,” RXXR (where X represents any amino acid), thus facilitating the entry of SARS-CoV-2 into host cells and increasing SARS-CoV-2 infectivity (Teesalu et al., 2009; Cantuti-Castelvetri et al., 2020). Meanwhile, NRP1 contributes to the tissue tropism of SARS-CoV-2 (Kyrou et al., 2021). Indeed, the upregulated gene expression of NRP1 was found in respiratory and olfactory epithelial cells of patients with COVID-19, which is associated with prominent symptoms, especially pulmonary and neurological manifestations (Gudowska-Sawczuk and Mroczko, 2021; Mayi et al., 2021). Therefore, blocking NRP1 may not only reduce the infectivity of SARS-CoV-2 but also prevent multisystemic diseases. Nevertheless, a single NRP1 inhibitor may not provide excellent antiviral efficacy (Cantuti-Castelvetri et al., 2020). Some single NRP1-targeting agents against COVID-19 have been developed, while almost all of them were still in preclinical studies (Perez-Miller et al., 2021; Kolarič et al., 2022).

**FIGURE 1:**
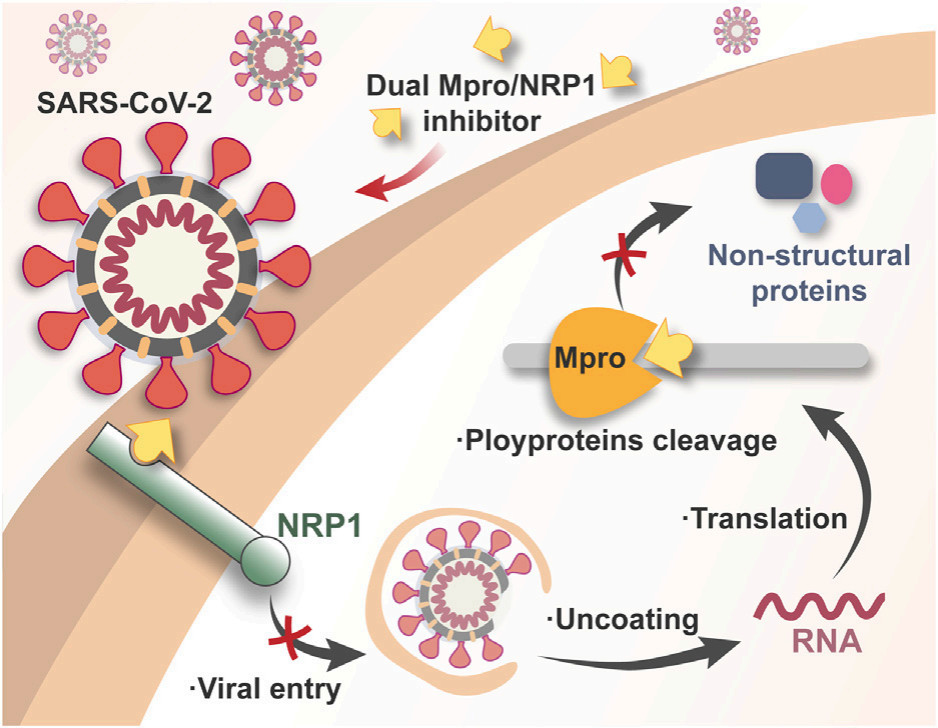
Schematic illustration of the mechanism of dual Mpro/NRP1-targeting inhibitors against SARS-CoV-2.

Simultaneously targeting Mpro/NRP1 by one entity is a promising and novel COVID-19 therapeutic that nearly no reported. In this way, by non-covalent inhibition of Mpro, we may block SARS-CoV-2 replication without the potential toxicity caused by covalent binding, while simultaneous inhibition of NRP1 may achieve the synergetic antiviral effect through the blockade of SARS-CoV-2 entry (Figure 1). Importantly, as two less mutable targets, dual Mpro/NRP1-targeting inhibitors may be effective against the variant Omicron BA.2.75 and even emerging SARS-CoV-2 VOCs in the future.

Cyclic peptides are an important class of drugs due to their splendid binding affinity and specificity (Vinogradov et al., 2019; Kreutzer et al., 2021). Compared with small molecule drugs, cyclic peptides are more biocompatible and can bind to large and open active pockets with strong interactions (Zhou et al., 2022). Moreover, compared with linear analogues, cyclic peptides often exhibit enhanced conformational stability, which leads to increased resistance to degradation by endogenous proteases, contributing to more efficient delivery *in vivo* (Qian et al., 2017). Herein, non-covalent cyclic peptides simultaneously targeting Mpro and NRP1 were retrieved through a structure-based virtual screening strategy that combines pharmacophore modeling, molecular docking, and molecular dynamics (MD) simulation. Then, they were purchased for validation in a series of bioassays. MN-2 showed the highest binding affinity to both Mpro and NRP1. Importantly, MN-2 exhibited significant inhibitory activity against Mpro and remarkable anti-infective activity against the pseudotyped variant Omicron BA.2.75 with undetectable cytotoxicity.

## Materials and methods

### Materials

Human embryonic kidney 293T (293T) cells, human lung alveolar epithelial (A549) cells, and human fetal small intestinal epithelial (FHs 74 Int) cells were obtained from American Type Culture Collection (ATCC) (Manassas, VA, United States). All cell lines were cultured in Dulbecco’s modified Eagle’s medium (DMEM) with 1% penicillin-streptomycin and 10% fetal bovine serum (FBS) in a humidified atmosphere, 5% CO_2_ at 37°C. All the peptides were purchased from GL Biochem (Shanghai, China). Mpro and NRP-1 were purchased from Abcam (Cambridge, MA, United States). The SARS-CoV-2 pseudovirus was obtained from ACROBiosystems (Newark, DE, United States). Peptide-21 and EG3287 were purchased from GL Biochem (Shanghai, China).

### Establishment of the Mpro pharmacophore model

A crystal structure of Mpro co-crystallized with a cyclic peptide inhibitor (PDB ID: 7RNW) was retrieved from the Protein Data Bank (PDB) database (http://www.rcsb.org). (Johansen-Leete et al., 2022) Then, it was loaded into the Molecular Operating Environment program (MOE, Chemical Computing Group Inc., Montreal, Quebec, Canada) for the following processing (chemcomp, 20222022). The Structure Preparation Tool was used to correct structure errors. Subsequently, the prepared structure was analyzed using the Surfaces and Maps tool and the Ligand Interactions tool. Based on the cleft-shaped active site of Mpro and key hydrogen-bond interactions between Mpro and the inhibitor, the Mpro pharmacophore model was manually established by the Pharmacophore Query Editor. Every feature in the Mpro pharmacophore model corresponded to crucial residues that formed hydrogen bonds, resulting in constraints on the amino acids of peptides for docking.

### Construction of the peptide database

The QuaSAR-CombiGen module of the MOE program was used to generate a fully-enumerated combinatorial library from a set of peptide fragments (Zhou et al., 2022). In this study, the QuaSAR-CombiGen enumerated a virtual library of all peptides that were combinatorially generated from two peptide fragments including cyclic peptides (containing 20 or 21 amino acids) and tetrapeptides (including RXXR motif). The oxygen atom on the hydroxyl group at the C-terminal end of each cyclic peptide was labeled as the “A1” port, while the hydrogen atom on the N-terminal end of each tetrapeptide was labeled as the “A0” port. The entire combinatorial library was enumerated by exhaustively cycling through all combinations of cyclic peptide fragments at the attachment “A1” port and tetrapeptide fragments at the attachment “A0” port. The virtual library containing 20,000 cyclic peptides was written to an output database.

### Docking-based virtual screening

The 2D database with a total of 20,000 cyclic peptides was used for docking-based virtual screening. The Energy Minimization protocol of MOE was applied to convert 2D peptides into 3D peptides. To screen out Mpro-targeting peptides from the 3D peptide database, the Dock program of MOE with the Mpro pharmacophore model as position constraints was utilized (Zhou et al., 2022). The Docking Scoring function of MOE was then employed to calculate binding free energy between peptides and Mpro, and potential Mpro-targeting peptides were selected with a rational docking score threshold for the subsequent filtration of simultaneous NRP1-targeting peptides.

A crystal structure of the NRP1-BD (PDB ID: 7JJC) co-crystallized with the CendR peptide of the SARS-CoV-2 S1 protein was retrieved from the PDB database and pretreated and analyzed using the same tools as Mpro (Daly et al., 2020). Given that peptides with an arginine-rich C-end can effectively bind to the comparatively narrow active pocket of NRP1, a docking against the b1 domain without restrictions of the pharmacophore model was directly implemented on potential Mpro-targeting peptides. The docking screening was also performed by the Dock program of MOE. Finally, four cyclic peptides with dual Mpro/NRP1-targeting potential were screened out by defining a reasonable cut-off for docking scores computed by the Docking Scoring function.

### Molecular dynamics simulation

After docking, Mpro-peptide and NRP1-peptide complexes with the best binding poses were obtained and followed by MD simulations using GROMACS packages with an AMBER99SB-ILDN force field (Lindorff‐Larsen et al., 2010; Gromacs, 2022). These complexes were placed in a 1.0 nm diameter cubic box and solvated with extended simple point charge (SPC/E) water molecules (Berendsen et al., 1987). Sodium ions (Na^+^) and chloride ions (Cl^+^) were added to neutralize the systems. The neutralized systems were then energy minimized by the steepest descent method. The 100 ps NVT and NPT equilibrations were carried out under 1 bar and 300 K, respectively. Finally, 100 ns MD simulations were performed storing the conformation every 100 ps. RMSD and RMSF analysis were done on MD trajectory files.

### Microscale thermophoresis analysis

The MST experiments were conducted with a Monolith NT.115 instrument in buffer (pH 7.0) containing 50 mM Tris, and 230 mM NaCl (Qin et al., 2016). Mpro and NRP1 were labeled using the Lys labeling kit for detection in the MST experiments. The final concentration of either labeled protein in the assay was 50 nM. According to manufacturer’s recommendations, the tested peptides were titrated in 1:1 dilution starting from 12.5 μM (according to the solubility of each peptide). Then, each binding reaction was centrifuged at 15,000 rpm for 5 min and loaded into standard glass capillaries for the MST analysis. All measurements were conducted using automatically assigned 20% LED and 50% MST power.

### Enzymatic assay

According to a previously reported method (Du et al., 2021), the FRET-based enzymatic assay was used to evaluate the inhibitory effects of the peptides on Mpro. First, the Mpro (250 nM at a final concentration) was incubated with various concentrations of tested peptides in 90 μL reaction buffer for 30 min in a black 96-well plate, and then the reaction was initiated by adding 10 μL of 50 nM FRET-based peptide substrate (Dabcyl-KTSAVLQ/SGFRKME-Edans). The reaction was monitored for 1 h, and the initial velocity was calculated using the data by linear regression. The IC_50_ was calculated by plotting the initial velocity against various concentrations of testing inhibitor by using a four parameters dose−response curve in Prism software.

### Pseudovirus infection and treatment

According to a previously reported method (Zheng et al., 2021a), the SARS-CoV-2 pseudovirus Omicron BA.2.75 was used to assess the infection-blocking effect of peptides on the virus. The pseudovirus is derived from the pseudotyped HIV-1 virus expressing the SARS-CoV-2 Omicron BA.2.75 spike protein on the surface and contains the firefly luciferase reporter gene for detection; hence, when the pseudovirus enters the host cells, the luciferase would be expressed. The 293T cells were seeded at a density of 2 × 10^4^ cells per well in 96-well plates at 37°C overnight. The cells were incubated with peptides for 1 h at 37°C and were then added to a titer of pseudovirus (relative luminescence units ranging from 20,000 to 40,000). After being cultured for 2 days, the cells were harvested by cell lysis buffer containing the luciferase detection reagent and detected by a luciferase detection kit according to the manufacturer’s instructions (Promega).

### Cytotoxicity assay

According to a previously reported method (Zheng et al., 2021b), 293T, A549 and FHs 74 Int cells were plated in 96-well plates at a density of 5 × 10^4^ cells per well. After incubation for 24 h, the peptide solution was added into the plates and the cells were incubated for 48 h. After that, the incubation medium was removed, the cells were washed with PBS at pH 7.4, and then MTT stock solution (0.5 mg/ml) was added into each well and incubated for another 4 h. The medium was removed by centrifugation, and the precipitated cells were lysed using DMSO. After the purple crystals were completely dissolved, the cell viability was calculated according to the absorbance signals measured by a microplate reader at the wavelength of 570 nm.

### Circular dichroism spectroscopy

The CD spectra were recorded in the far-UV range from 190 to 250 nm in optical cell with a path length of 0.1 cm (JASCO J-180) at 25°C (Rawat et al., 2010). The concentration of MN-2 solution was adjusted to 0.5 mg/ml in PBS (pH 7.4).

### Statistical analysis

All results were expressed as the mean ± SD. Statistical analysis was performed with the *t*-test for two groups. Significance levels at *p* < 0.05 and 0.01 were considered to indicate statistical significance.

## Results and discussion

### Virtual screening for dual Mpro/NRP1-targeting peptides

**FIGURE 2:**
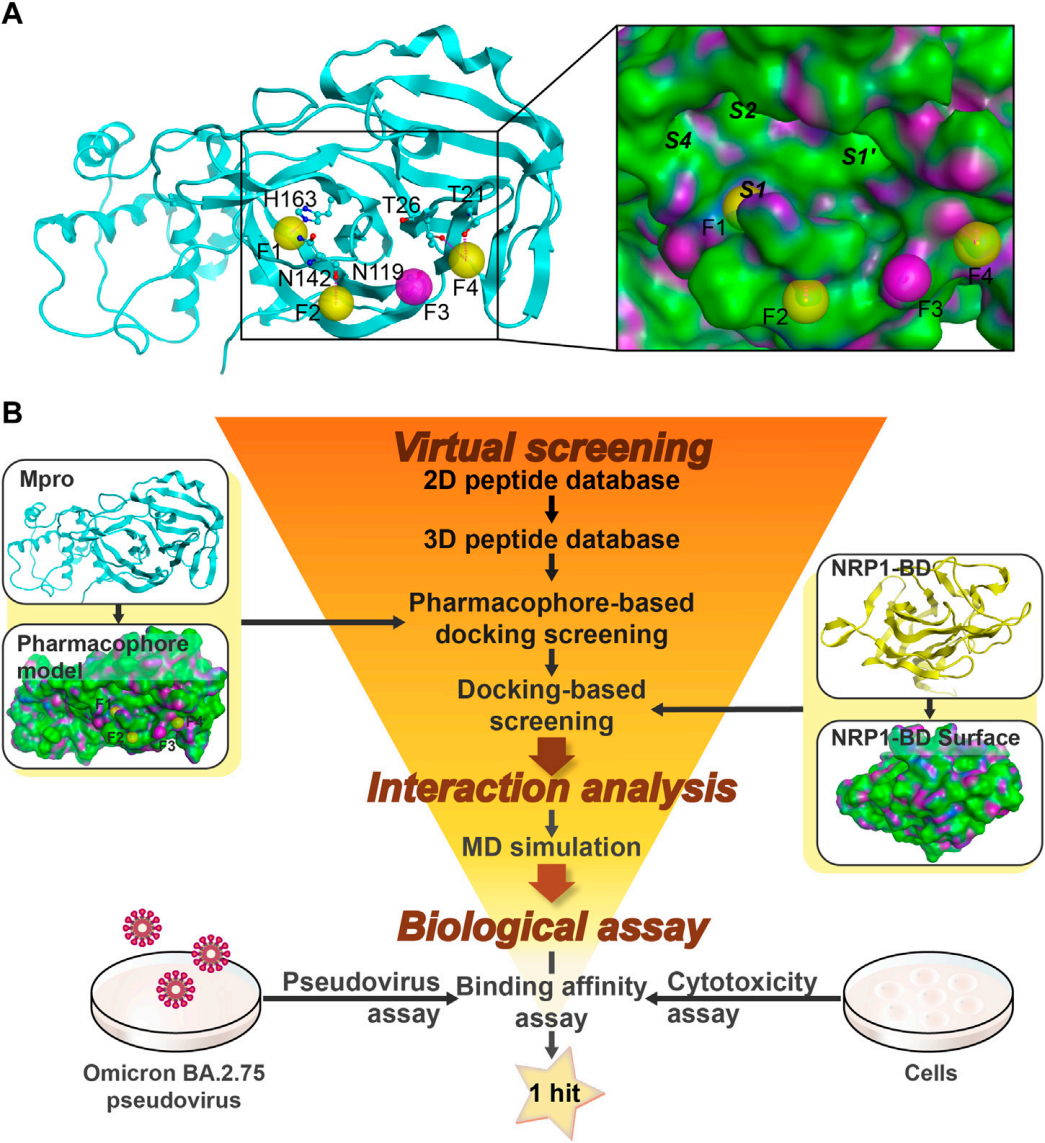
Identification of cyclic peptides targeting Mpro/NRP1. **(A)** Details of the Mpro-based pharmacophore model (F1, F2, and F4 are hydrogen-bond donor features, and F3 is a hydrogen-bond acceptor feature). **(B)** The flowchart of virtual screening, interaction analysis, and biological assay for identification of dual Mpro/NRP1-targeting peptides. Pharmacophore features of Mpro are represented as spheres. Hydrogen bonds are represented by purple dotted lines. Italics indicate Mpro sub-pockets. Protein secondary structures are shown in line form. The surface of the Mpro and NRP1-BD are plotted by H-bonding (purple), hydrophobicity (green), and mild polar (blue) regions.

A crystal structure of Mpro co-crystallized with a cyclic peptide inhibitor (PDB ID: 7RNW) was retrieved to provide insights into the establishment of a Mpro pharmacophore model. As described above, Mpro exerts proteolytic activity as a dimer composed of two protomers. Each protomer comprises three domains (Domain I, II, and III), and the substrate-binding site containing the C145-H41 catalytic dyad is located in a wide cleft between Domains I and II (Zhao et al., 2022). As can be seen in the inset of Figure 2A, the wide cleft can accommodate Mpro substrates in vital sub-pockets, such as S1’, S1, S2, and S4, where cleavage occurs between P1-P1’ of the substrates (corresponding to S1-S1’) (Cannalire et al., 2020; Deshmukh et al., 2021). Lots of peptidomimetic inhibitors imitated the substrate sequence L-Q-(S, A, G) (L at the S2 position and Q at the S1 position) to bind tightly to the Mpro active site, blocking Mpro by forming key interactions, including hydrogen-bond interactions, hydrophobic interactions, or the covalent bond with C145 (Pillaiyar et al., 2016; Johansen-Leete et al., 2022). Herein, to avoid the potential toxicity of covalent inhibitors, four pharmacophoric features (F1-F4) indicating the crucial hydrogen-bond interaction sites of hits bound to Mpro rather than the covalent binding site were created (Figure 2A). Considering that inhibitory activity requires vast occupation of the wide cleft, the four pharmacophoric features were set across the head and tail of the cleft, the latter three of which are located at the outer edge of the cleft to maintain rigid cyclic peptides in excellent conformation poses for better insertion into the cleft (Figure 2A). Except for the feature F3, which was characterized as a hydrogen-bond acceptor, the rest three features were hydrogen-bond donors. The feature F1 corresponded to the critical residue H163 which is a component of the sub-pocket S1 (Nguyen et al., 2020). The feature F2 corresponded to residue N142, and the feature F3 corresponded to residue N119. The last feature F4 corresponded to two residues T26 and T21. The final Mpro pharmacophore model was constructed from the features F1-F4 and prepared for docking screening of Mpro-targeting non-covalent cyclic peptides (Figure 2A).

A workflow of multistep virtual screening in this study is presented in Figure 2B. The two-dimensional (2D) database with 20,000 total cyclic peptides was energy-minimized in preparation for the 3D database. The Mpro pharmacophore model was then used as a restriction to dock against the wide cleft of Mpro. After setting a rational docking score threshold of < −13.4 kcal/mol, a total of 248 potential Mpro-targeting peptides were obtained.

It was found that the amino acid sequence RRAR (which conforms to “CendR”) of the SARS-CoV-2 S protein was able to bind to the NRP1-BD. Therefore, the high-resolution crystal structure of NRP1-BD (PDB ID: 7JJC) was retrieved and used as the receptor docked next. The active pocket of the b1 domain is a relatively narrow depression that most peptides with arginine at the C-end can bind to it *via* hydrogen-bond interactions. In light of this, direct docking of the previously obtained Mpro-targeting peptides into the active pocket of the b1 domain can be employed as a filtration criterion for NRP1-targeting peptides. Based on docking scores below −12.1 kcal/mol (Supplementary Table S1), four potential dual Mpro/NRP1-targeting peptides were finally screened out. To further investigate interactions between these four peptides and Mpro/NRP1, a combined docking and MD simulation method was subsequently implemented (Figure 2B).

### Interaction analysis

**FIGURE 3:**
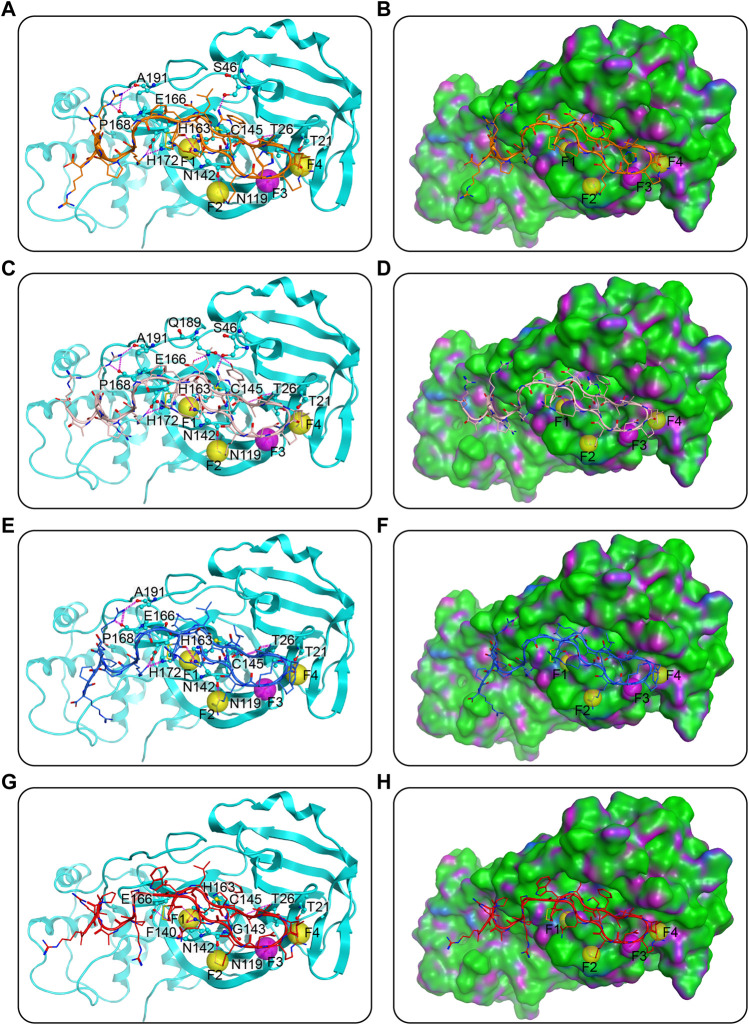
Predicted docking poses of MNs 1-4 at the Mpro active site. **(A)**, **(C)**, **(E)**, and **(G)** are MNs 1-4, respectively, and **(B)**, **(D)**, **(F)**, and **(H)** are their corresponding surface plots. Peptides are represented by different colors (orange for MN-1, pink for MN-2, blue for MN-3, and red for MN-4), and Mpro is color-coded by cyan-blue. Pharmacophore features of Mpro are represented as spheres. The hydrogen bonds were indicated by purple dotted lines. The surface of the Mpro is plotted by H-bonding (purple), hydrophobicity (green), and mild polar (blue) regions.

Docking can assist in predictions of binding poses. The four potential dual Mpro/NRP1-targeting peptides were docked into active sites of Mpro and NRP1 successively. As shown in Figure 3, each cyclic peptide contains a ring part (head) which was formed by a disulfide bond at the N-end, an arginine-rich part (corresponding to the “C-end rule” RXXR), and a linker, the latter two constituting the ringless part (tail). The ring head of each peptide occupied most of the cleft in Mpro, and hydrogen-bond interactions created by it were a major contributor to the binding of peptides to Mpro (Figures 3A,C,E,G). The second to fifth amino acids at the N-end of the peptides, named P3, P2, P1, and P1’, were well inserted into sub-pockets S4, S2, S1, and S1’, respectively (Figures 3B,D,F,H). Among them, the P2 position was amino acid L and the P1 was Q, which were identical to the Mpro substrates, while both positions P3 and P1’ were hydrophobic amino acids. Specifically, the amino acid at position P3 was well positioned in hydrophobic sub-pocket S4, and was in D conformation for the prevention of collision with sub-pocket S2; L at position P2 inserted deeply into the hydrophobic sub-pocket S2; the side chain of the amino acid Q at position P1 was mapped onto the pharmacophoric feature F1, forming a hydrogen-bond with residue N142 besides the other with the pharmacophore’s corresponding residue H163, and importantly, the backbone carbonyl oxygen of Q also formed a hydrogen-bond with the catalytic key residue C145; and the hydrophobic amino acid at position P1’ was located well at the hydrophobic sub-pocket S1’ (Figures 3). The sequence L-Q may enable the peptides to be recognized by Mpro, and the sequence with hydrophobic side chains may stabilize the binding to hydrophobic sub-pockets by engaging in hydrophobic interactions. Amino acids outside the P3-P2-P1-P1’ sequence of the ring head were involved in hydrogen-bond interactions with residues N142, N119, T26, and T21 of Mpro, which corresponded to features F2-F4. Outside the features, additional hydrogen bonds with residues S46 and E166 in Mpro cleft were formed by those amino acids; the former was only in MN-1 and MN-2, while the latter was only on the N-terminal cysteine of MN-2 and MN-4. Although mainly the ring heads of peptides dominated the Mpro cleft, the rest parts also made some contributions to the binding of Mpro. The linkers of MN-1 and MN-3 were found to have hydrogen-bond interactions with residues E166 and H172. The hydrogen bond with residue H172 was also shown in the linker of MN-2, whereas absent in MN-4. Furthermore, the C-end arginine-rich parts of MNs 1-3 were stabilized by three hydrogen bonding interactions with residues A191 and P168, which were yet not present in MN-4. Besides, MN-4 had one hydrogen bond that was not observed in all the other three peptides, which was formed with residue N140 of Mpro by the side chain of amino acid Q at position P1.

**FIGURE 4:**
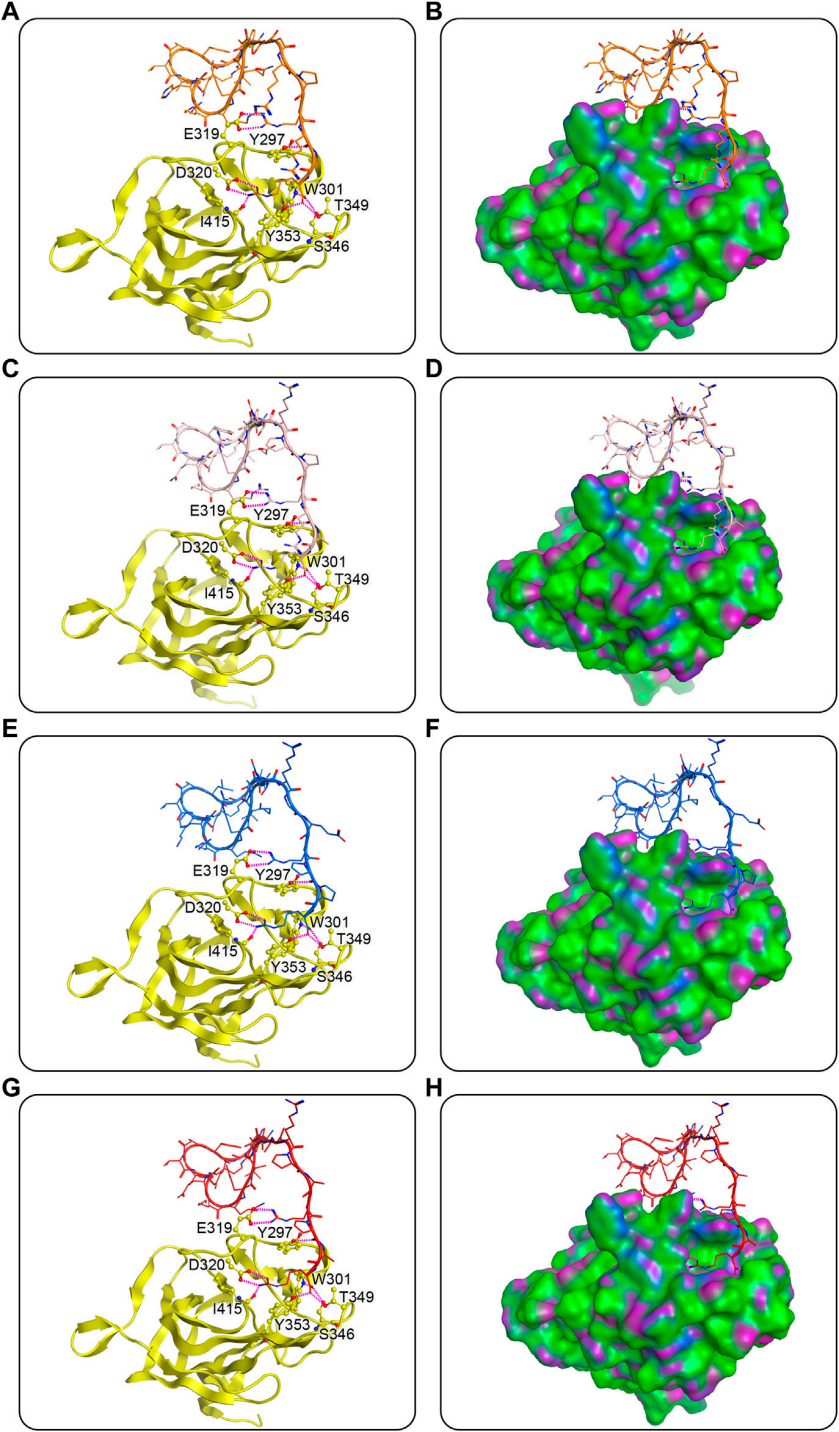
Predicted docking poses of MNs 1-4 at the NRP1-BD. **(A)**, **(C)**, **(E)**, and **(G)** are MNs 1-4, respectively, and **(B)**, **(D)**, **(F)**, and **(H)** are their corresponding surface plots. Peptides are represented by different colors (orange for MN-1, pink for MN-2, blue for MN-3, and red for MN-4), and NRP1-BD is color-coded by yellow. The hydrogen bonds were indicated by purple dotted lines. The surface of NRP1-BD is plotted by H-bonding (purple), hydrophobicity (green), and mild polar (blue) regions.

In the case of docking against NRP1, predicted interactions are depicted in Figure 4. Predominantly the C-end arginine-rich part (i.e., peptide sequence RXXR) of the peptides formed hydrogen-bond interactions with critical residues of the active pocket of NRP1, namely E319, Y297, D320, W301, T349, I415, T353, and S346 (Figures 4A,C,E,G). The last amino acid arginine of MNs 1-4 extended into the active pocket featuring seven hydrogen bonds with residues D320, W301, T349, I415, T353, and S346. The fourth from the end amino acid arginine formed two hydrogen bonds with residue E319 on the outer edge of the active pocket. The second to last and the third to last amino acids varied in every peptide, but the carbonyl oxygen of their backbones all formed a hydrogen bond with residue Y297. In the ring head and linker of the four peptides, there were no hydrogen-bond interactions with the NRP1-BD observed, suggesting that these parts were not the main force for binding to NRP1, which was as expected (Figures 4B,D,F,H).

### Molecular dynamics simulation

**FIGURE 5:**
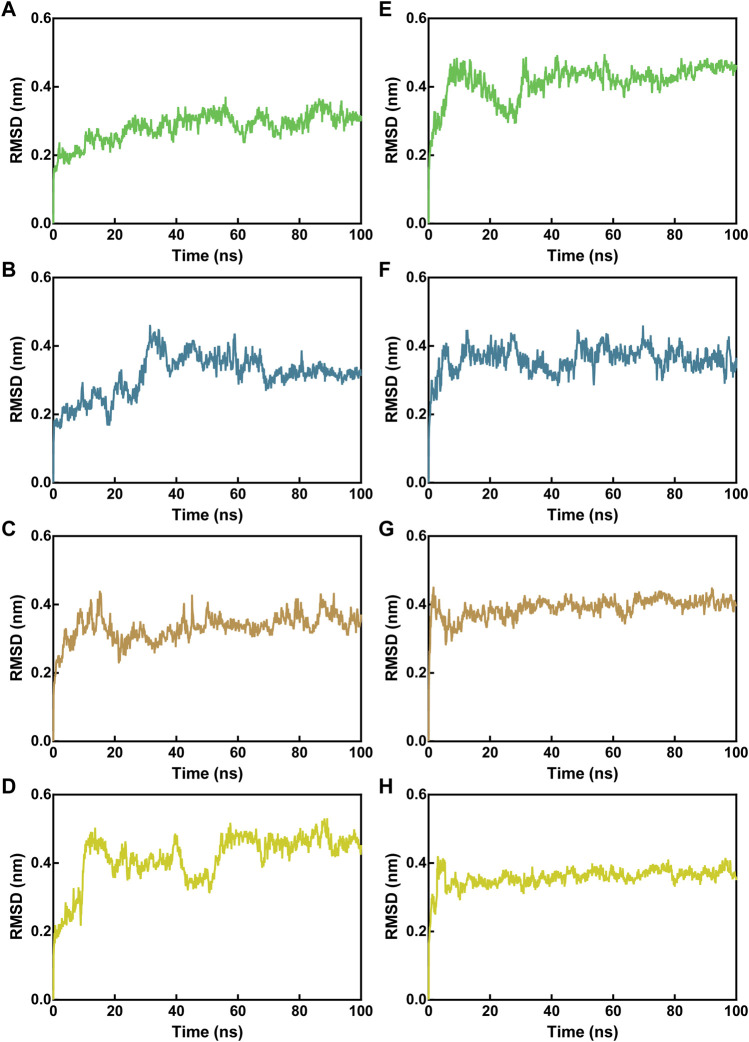
RMSD of Cα atoms of Mpro-peptide and NRP1-peptide complex atoms with respect to the initial structures obtained from docking. **(A–D)** RMSD in Mpro-peptide complexes, and **(E–H)** RMSD in NRP1-peptide complexes. In all panels the color code is MN-1 (green), MN-2 (blue), MN-3 (brown), and MN-4 (yellow).

To test the binding stability of MNs 1-4 at the active site of Mpro and NRP1 in a dynamic view, we performed 100 ns MD simulations. First, the root-mean-square deviations (RMSDs) of the Cα atoms in Mpro-peptide and NRP1-peptide complexes were calculated for analyzing the MD trajectory equilibration. Lower RMSD values tend to imply better binding stability. As depicted in Figure 5, all Mpro-peptide complexes reached equilibrium after 70 ns whereas NRP1-peptide complexes reached equilibrium after 50 ns, and their mean RMSD values for final equilibrium were below 0.5 nm. In Mpro-peptide systems, the mean RMSD values of Mpro-MN-1, Mpro-MN-2, Mpro-MN-3, and Mpro-MN-4 were 0.28, 0.31, 0.33, and 0.41 nm, respectively. The mean RMSD values for MN-1 and MN-2 were comparatively lower indicating better binding stability to Mpro. In the case of NRP1, the mean RMSD values of NRP1-MN-1, NRP1-MN-2, NRP1-MN-3, and NRP1-MN-4 were 0.41, 0.36, 0.39, and 0.36 nm, respectively, suggesting that MN-2 and MN-4 may have relatively better binding stability to NRP1 than MN-1 and MN-3.

**FIGURE 6:**
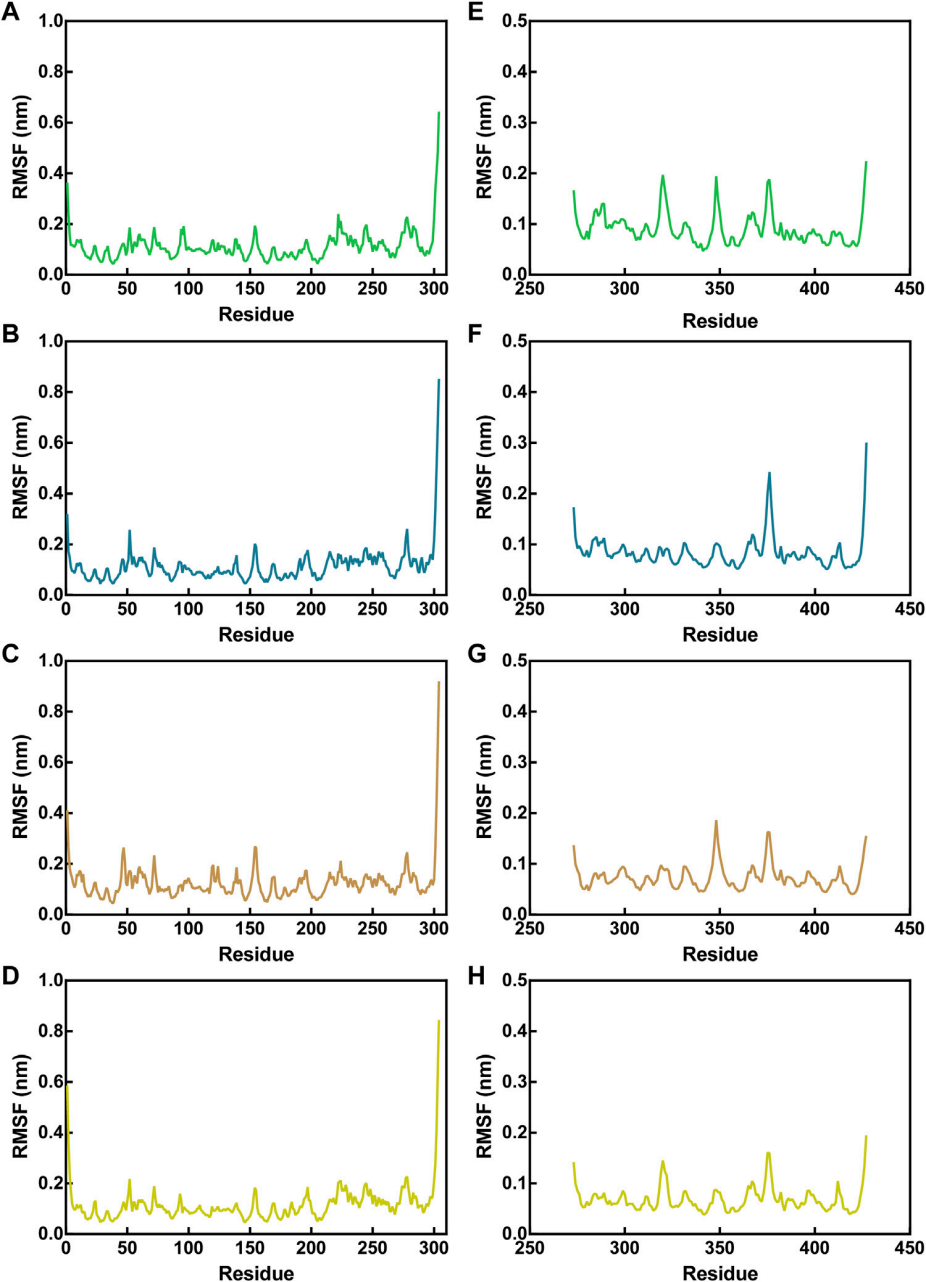
RMSF of Cα atoms of Mpro residues in Mpro-peptide complexes **(A–D)** and NRP1 in NRP1-peptide complexes **(E–H)**. In all panels the color code is MN-1 (green), MN-2 (blue), MN-3 (brown), and MN-4 (yellow).

The flexibility of amino acid residues within Mpro and NRP1 in the complex systems was reflected by assessing the root-mean-square fluctuations (RMSFs) of Cα atoms. Lower RMSF values indicate fewer residue movements, which tend to be correlated with some interactions between Mpro/NRP1 and peptides, such as hydrogen-bond interactions. For Mpro-peptide complexes, the RMSF values of Mpro residues in the presence of different peptides were nearly identical, indicating similar binding stability of these peptides to Mpro (Figures 6A–D). The residues corresponding to pharmacophoric features of the Mpro pharmacophore model, i.e., T21, T26, N119, N142, and H163, showed limited fluctuations with RMSF values below 0.2 nm. In addition, the residues of the other hydrogen bonds formed with Mpro, such as residues C145 and E166, exhibited small RMSF fluctuations in the range of <0.1 nm. The largest fluctuations of Mpro residues occurred at the C-/N-ends, which was associated with no interactions they generated with the peptides. In NRP1-peptide simulations, the degree of residue deviation in every complex system was small, even at the C-/N-ends (Figures 6E–H). The NRP1-BD residues predicted to form hydrogen bonds during NRP1-BD docking with peptides, including NRP1-BD resides E319, Y297, D320, W301, T349, I415, T353, and S346, all had low RMSF values below 0.2 nm. As shown in Figures 6A,E–H relatively larger RMSF fluctuation of NRP1-BD crucial residue D320 was found in MN-1 and MN-4, while its fluctuation value was lowest in MN-2. In the NRP1-MN-2 complex, except for the comparatively more flexible residue at C-end, the NRP1-BD residue N376 also displayed an RMSF fluctuation of >0.2 nm (Figure 6F). However, this residue N376 is located away from the binding site and its greater flexibility may be due to the lack of potential interactions of MN-2 with it. Taken together, these docking and MD findings revealed that MNs 1-4 have the potential to interact with critical active-site residues of Mpro and NRP1 with good binding stability.

### Identification of peptides targeting Mpro and NRP1

**FIGURE 7:**
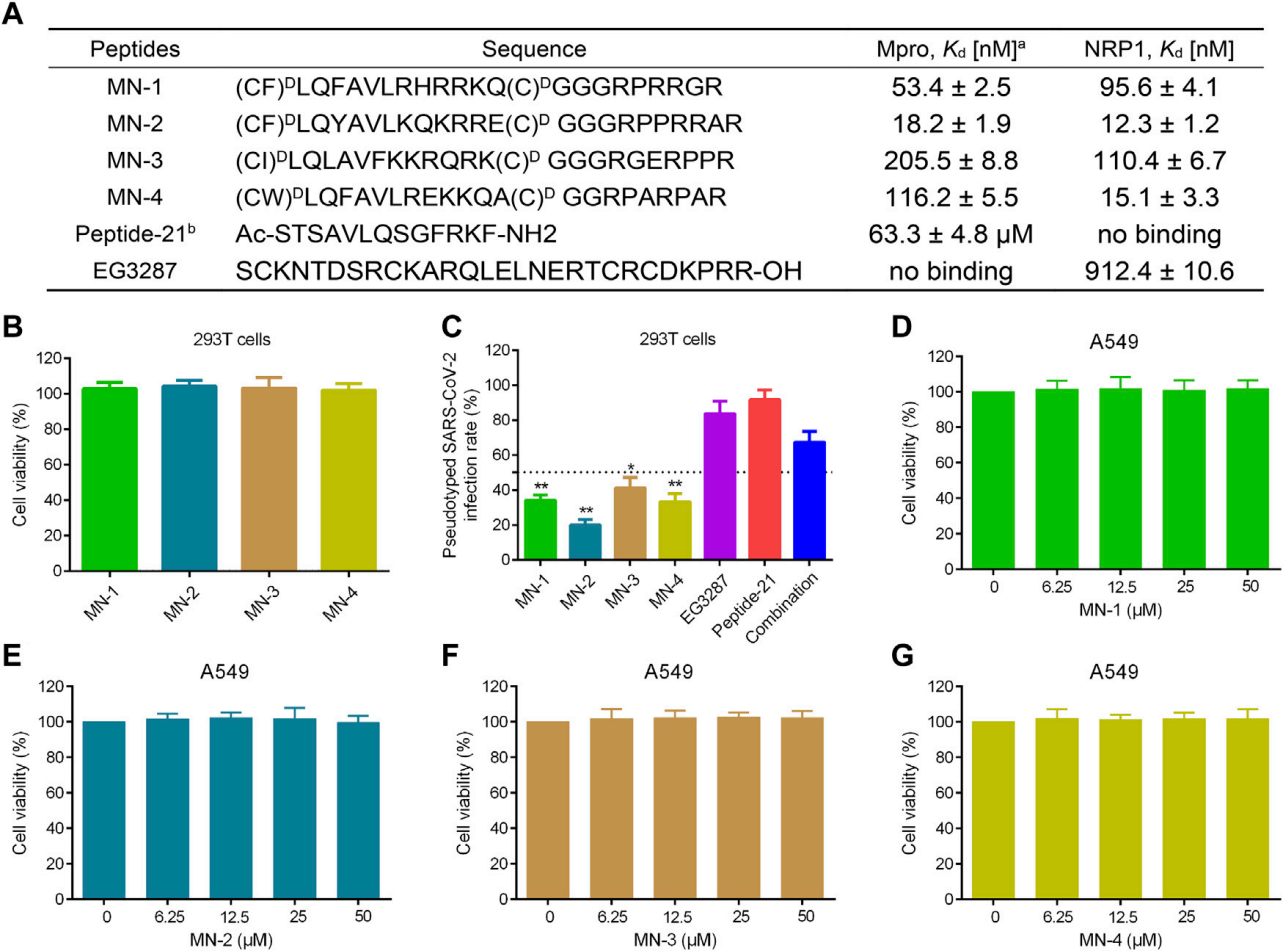
Binding affinity, anti-pseudovirus infection, and cytotoxicity of MNs 1-4. **(A)** Sequences and binding affinities of peptides. ^a^MST data shown represent the mean ± SD (*n* = 3). ^b^Peptide-21 and EG3287 served as the positive controls. MNs 1-4 were cyclized through a disulfide bond formed by two cysteines. **(B)** The cytotoxicity effects of MNs 1-4 on 293T cells using MTT assay. **(C)** Infection rate of the screened peptides MNs 1-4, EG3287, Peptide-21, and the combination of EG3287 and Peptide-21 against pseudotyped SARS-CoV-2 Omicron BA.2.75 at a concentration of 2 μM in 293T cells. **(D**–**G)** The cytotoxicity effects of MNs 1-4 on A549 cells detected using MTT assay. Cells were treated with different concentrations (0–50 μM) of peptides for 48 h. The results are represented as mean ± SD (*n* = 3). *p < 0.05, **p < 0.01 means a significant difference *versus* Peptide-21.

The binding affinity of MNs 1-4 to Mpro and NRP1 was evaluated using the microscale thermophoresis (MST) method. MNs 1-4 showed strong binding affinities to both Mpro and NRP1 with dissociation constants (*K*_d_) values ranging from 18.2 to 205.5 nM and 12.3–110.4 nM, respectively (Figure 7A). Of these, MN-2 had a highly potent binding affinity, exhibiting the best *K*_d_ values for Mpro (*K*_d_ = 18.2 ± 1.9 nM) and NRP1-BD (*K*_d_ = 12.3 ± 1.2 nM). Furthermore, the binding affinity of Peptide-21 (a positive inhibitor targeting Mpro) and EG3287 (a positive inhibitor targeting NRP1) to Mpro and NRP1 was evaluated, respectively (Jia et al., 2006; Ullrich et al., 2021). As can be seen in Figure 7A, the two positive inhibitors showed single good binding affinity to corresponding targets. Peptide-21 had a binding affinity for Mpro with a *K*_d_ value of 63.3 ± 4.8 μM while having no binding affinity for NRP1, and EG3287 had a binding affinity for NRP1 with a *K*_d_ value of 912.4 ± 10.6 nM while having no binding affinity for Mpro. Unlike Peptide-21 and EG3287, MN-2 exhibited dual binding affinity to Mpro and NRP1, which was about 3,478-fold and 74-fold stronger than that of the two positive inhibitors, respectively.

### Inhibitory effects of MNs 1-4 on Mpro

To evaluate the effect of MNs 1-4 on the enzymatic inhibition of SARS-CoV-2 Mpro, the FRET-based Mpro enzymatic assay was performed. The Mpro-targeting inhibitor Peptide-21 mentioned above was included as a positive control. As can be seen in Supplementary Table S2, the IC_50_ values of MNs 1-4 were 56.3 ± 3.9, 20.6 ± 2.2, 208.7 ± 7.6, and 119.4 ± 6.1 nM, respectively, which were all lower than that of Peptide-21 (IC_50_ = 68.2 ± 5.5 μM). These values suggested that MNs 1-4 have significant inhibitory activity against Mpro.

### Anti-infective efficacy of peptides

Pseudotyped SARS-CoV-2 is safe and easy to manipulate experimentally compared with high infectivity and pathogenicity of live SARS-CoV-2 (Chen and Zhang, 2021; Neerukonda et al., 2021; Xiang et al., 2022). Therefore, to evaluate the anti-infective efficacy of the screened peptides MNs 1-4, pseudovirus anti-infection assays were conducted. Firstly, MNs 1-4 at a 30 μM concentration displayed no significant effect on the viability of 293T cells in cytotoxicity assays (Figure 7B). Pseudotyped SARS-CoV-2 anti-infection assays were followed then under this non-toxic concentration. As shown in Figure 7C, MNs 1-4 exerted potent anti-infective activity against the variant Omicron BA.2.75 pseudovirus at a concentration of 2 μM, which was stronger than that of Peptide-21 or EG3287 alone or even their combination. Among them, MN-2 was found to have the most potent anti-infective activity with about 80% inhibition rate of the variant Omicron BA.2.75, indicating its highest anti-infective potential. Notably, compared with the combination of Mpro- and NRP1-targeting positive inhibitors, stronger anti-infective rates were observed in MN-2, which suggested a potential synergistic effect. Further validation in human lung alveolar epithelial (A549) cells also revealed significant anti-infective activity of MN-2 (Supplementary Table S1).

### Cytotoxicity of peptides

As mentioned above, the expression of entry receptors was found to be upregulated in the respiratory cells of COVID-19 patients, whose pulmonary manifestations are the main symptoms (Schurink et al., 2020). Hence, the exploration of the toxicity of peptides MNs 1-4 in A549 cells was further carried out. In MTT assays, A549 cells were treated with different concentrations (0, 6.25, 12.5, 25, 50 μM), and their viability was not affected by MNs 1–4 (Figures 7D–G). Also, the viability of normal cells, human fetal small intestinal epithelial (FHs 74 Int) cells, was not affected by MNs 1-4 at a concentration of 50 μM (Supplementary Figure S2). Even at such a high concentration of 50 μM, MN-2 still showed undetectable cytotoxicity, demonstrating its superior safety. Collectively, these results indicated that MN-2 is a highly potent dual-targeting agent against the variant Omicron BA.2.75 without obvious toxicity. In addition, to examine the conformational changes of MN-2, the CD spectra were measured (Supplementary Figure S3). The result showed a negative peak at 198 nm, attributed to the random coil conformation.

## Conclusion

The Omicron variant is the most distinct variant exhibiting the highest degree of immune evasion to current COVID-19 vaccines and causing unprecedented infections and deaths (Cao et al., 2022; Gobeil et al., 2022). Lack of efficacy of available vaccines against Omicron, especially its sub-variant BA.2.75, has urged the development of novel therapeutics. Notably, specifically targeting less mutable targets may be successful in bypassing the immune evasion resulting from continuous mutations on the S protein of SARS-CoV-2. As we know, SARS-CoV-2 Mpro and human NRP1 as two less mutable proteins play a pivotal role in SARS-CoV-2 replication and entry into host cells, respectively. Therefore, our study, based on a novel therapeutic strategy that non-covalently targeting Mpro and NRP1, identified four cyclic peptides using pharmacophore model, molecular docking, and MD simulation. These four peptides mainly formed hydrogen bonds rather than covalent bonds to block Mpro and NRP1 with nanomolar range binding affinity. Among these, MN-2 exhibited the most potent binding affinity and was several orders of magnitude higher than that of positive inhibitors. Further evidence demonstrated that MN-2 have significant inhibitory activity against Mpro and remarkable anti-infective activity against the pseudotyped variant Omicron BA.2.75 without detectable cytotoxicity.

It is however, worth mentioning that peptide MN-2 still needs further activity evolution and *in vivo* validation. The cyclic peptide MN-2 formed by a disulfide bond may be liable to break in response to reducing agents in plasma, resulting in a reduction of its biological activity and utility as a therapeutic agent (Muttenthaler et al., 2010a; Olson et al., 2016). To solve this potential issue, alternative cyclization strategies can be explored, including dicarba, lactam, and diselenide (Hargittai et al., 2000; Macraild et al., 2009; Muttenthaler et al., 2010b). As mentioned above, the ring head of peptide MN-2 occupied most of the cleft in Mpro and the hydrogen-bond interactions formed by this part were a major contributor to binding, while as can be seen in Figure 3D, the hydrophobic surface, such as the sub-pockets S4 and S1’, tolerates more modification to enhance hydrophobic interactions. Notably, although MN-2 showed significant activity in both biochemical assays and pseudovirus-based cell assays, the antiviral efficacy of MN-2 still requires testing in available preclinical animal models of SARS-CoV-2.

In summary, based on above range of experimental studies, MN-2 is a novel non-covalent cyclic peptide with a highly potent dual-targeting efficacy, which could act as a potential antiviral inhibitor against Omicron BA.2.75 and even emerging SARS-CoV-2 VOCs in the future.

## Data Availability

The original contributions presented in the study are included in the article/Supplementary Material, further inquiries can be directed to the corresponding authors.
