# Supplementary material for: Non-covalent cyclic peptides simultaneously targeting Mpro and NRP1 are highly effective against Omicron BA.2.75
Source: Front Pharmacol. 2022 Nov 2;13:1037993. doi: 10.3389/fphar.2022.1037993 (PMC9666779; doi:10.3389/fphar.2022.1037993)
Supplement: Supplementary file 1 [file DataSheet1.docx]

Supplementary Material

**Non-covalent cyclic peptides simultaneously targeting Mpro and NRP1 are highly effective against Omicron BA.2.75**

**Table S1. The docking scores of the screened peptides**

|  | Mpro | NRP1-BD |
| --- | --- | --- |
| Name | Binding free energy*^a^* | Binding free energy |
|  | (kcal/mol) | (kcal/mol) |
| MN-1 | -13.74 | -12.15 |
| MN-2 | -13.79 | -13.63 |
| MN-3 | -13.44 | -12.11 |
| MN-4 | -13.58 | -13.59 |

*^a^*Binding free energy between the peptide and the target (lower binding free energies suggest stronger binding affinities).

**Table S2. Inhibitory effects of MNs 1-4 and Peptide-21 on Mpro**

|  | MN-1 | MN-2 | MN-3 | MN-4 | Peptide-21 |
| --- | --- | --- | --- | --- | --- |
| IC_50_ (nM) ± SD*^a^* | 56.3 ± 3.9 | 20.6 ± 2.2 | 208.7 ± 7.6 | 119.4 ± 6.1 | 68.2 ± 5.5 μM |

*^a^*The results are represented as mean ± SD (n = 3).

**

**

**Figure S1.** Infection rate of the screened peptides MNs 1-4, EG3287, Peptide-21, and the combination of EG3287 and Peptide-21 against pseudotyped SARS-CoV-2 Omicron BA.2.75 at a concentration of 2 μM in A549 cells. *P < 0.05, **P < 0.01 means a significant difference versus Peptide-21.

**

**

**Figure S2.** The cytotoxicity effects of MNs 1-4 on FHs 74 Int cells detected using MTT assay. Cells were treated with the concentration of 50 μM of peptides for 48 h. The results are represented as mean ± SD (n = 3).





**Figure S3.** The secondary structure of MN-2 monitored by CD spectroscopy.
